# Deciphering the Causality between Gut Microbiota Dysbiosis and Poisoning by Narcotics and Psychodysleptics: A Mendelian Randomization Analysis

**DOI:** 10.2174/1570159X22999240729092453

**Published:** 2024-07-30

**Authors:** Ning Wang, Zhenbo Su

**Affiliations:** 1 Department of Anesthesiology, China-Japan Union Hospital of Jilin University, Changchun, China;; 2 Department of Anesthesiology, Shanghai Ruijin Hospital, Shanghai, China

**Keywords:** Gut microbiota, mendelian randomization, narcotics, psychodysleptics, drug metabolism, adverse effect

## Abstract

**Background:**

This study investigates the connection between gut microbiota and poisoning caused by narcotics and psychodysleptics, using Mendelian randomization (MR) to explore possible causal relationships.

**Methods:**

The study employed the MR analysis, leveraging genetic variants as instrumental variables to facilitate robust causal inference. Data for gut microbiota was extracted from the MiBioGen study, integrating genome-wide genotyping data with 16S fecal microbiota profiles. Outcome metrics were based on the Finngen study. Genetic instruments were meticulously extracted based on stringent criteria, and harmonized with SNP outcomes associated with “Poisoning by narcotics and psychodysleptics (hallucinogens)”. The inverse-variance weighted (IVW) method was utilized for MR analysis, supplemented by sensitivity analyses including MR-Egger Regression, Weighted Median Approach, and Leave-One-Out Cross-Validation.

**Results:**

Among various microbial groups, nine showed significant statistical links. Specifically, Class Negativicutes (OR 5.68, 95% CI 2.13-15.16, *p* = 0.0005) and Order Selenomonadales (OR 5.68, 95% CI 2.13-15.16, *p* = 0.0005) were notably associated. These findings were consistent across different sensitivity analyses.

**Conclusion:**

The relationship between gut microbiota and the adverse effects of narcotics and psychodysleptics is an emerging area of research. Our MR study identifies certain microbes that might influence the body's response to these substances. These insights could help in predicting and treating the effects of narcotics and psychodysleptics in the future.

## 1 INTRODUCTION

Narcotics like morphine and fentanyl, which act on the central nervous system, are widely used for pain relief. The clinical application of narcotics is especially important for managing everything from short-term pain after surgery to long-term chronic pain [1]. Over one-third of the U.S. population contends with acute or persistent pain conditions, with the prevalence surpassing 40% among the elderly [2]. The widespread pain has led to an increasing reliance on narcotics such as opioids, solidifying their role as a primary prescription choice nationwide and highlighting their indispensable efficacy across various pain management scenarios [3, 4]. Psychodysleptics, or hallucinogens, like lysergic acid diethylamide (LSD) and psilocybin, alter perception and cognition [5, 6]. Beyond their historical recreational use, in controlled medical environments, they are increasingly finding applications in scenarios like the treatment of post-traumatic stress disorder (PTSD) [7, 8].

However, the widespread use of these drugs, along with cases of misuse, has led to increasing concerns [9]. Recent epidemiological data underscores an upward trajectory in both the prescription and misuse of opioids, with millions of prescriptions dispensed annually in the United States alone [10]. The extensive use of these substances, coupled with their potent pharmacological effects, results in a spectrum of adverse outcomes, commonly referred to as drug poisonings of narcotics and psychodysleptics [11-13]. This ranges from mild side effects, including dizziness, nausea, and altered mood to severe complications, such as hospitalization due to decreased oxygen saturation, respiratory depression, loss of consciousness, hospitalization and even fatalities [14-16]. Data from the Chinese National Disease Surveillance Points System, spanning from 2006 to 2016, illuminates a substantial escalation in unintentional fatal drug poisonings tied to narcotics and psychodysleptics among individuals aged 25-44. Specifically, rates surged from 0.4 to 0.7 per 100,000 among males and from 0.05 to 0.13 among females [17]. Beyond the individual health implications, the social and economic influences are far-reaching, underscoring the urgency of identifying risk factors associated with narcotics and psychodysleptics-related poisonings [18].

In recent years, the intricate interplay between gut microbiota and various health outcomes has drawn significant attention from researchers and clinicians [19]. The gut microbiome refers to the combined genetic material of all the microbes residing in the mammalian digestive system. It comprises more than 1000 species of bacteria and has a cell count that is approximately tenfold the number of host body cells [20]. Understanding the causal relationships between gut microbiota and specific health conditions, such as metabolic disorders [21], functional disorders [22], inflammatory diseases [23, 24] and even mental health [25], has become a central focus of biomedical research. The intricate relationship between gut microbiota and drug side effects has garnered cumulative attention in the scientific community [26, 27].

In this context, Mendelian randomization analysis, a robust method leveraging genetic variants as instrumental variables, has emerged as a powerful tool for investigating the causal links between gut microbiota composition and a range of health-related factors [28, 29]. This approach offers insights into a solid foundation for evidence-based interventions [30]. Recent studies have leveraged Mendelian randomization to elucidate the integral connections between gut microbiota and several diseases, underscoring its influence on diverse physiological processes [31-38].

In this study, we employ Mendelian randomization analysis to elucidate the causal relationships between gut microbiota and poisonings induced by narcotics and psychodysleptics, aiming to uncover potential connections and provide essential insights into the underlying mechanisms. Additionally, our objective is to identify clinical indicators capable of pinpointing individuals at increasing risk, with the ultimate goal of preventing poisonings brought by narcotics and psychodysleptics.

## 2 METHODS

### 2.1 Study Design and Data Source

In the intricate tapestry of biomedical research, the potential causal interplay between gut microbiota composition and susceptibility to poisoning by narcotics and psychodysleptics has emerged as a compelling narrative. To navigate this complex terrain, we employed the MR analysis, a cutting-edge methodology that exploits genetic variants as instrumental variables, thereby facilitating robust causal inference while adeptly circumventing confounders inherent to traditional observational studies. The design of this study was guided by the STROBE-MR guideline [39]. The overall flow chart of this study is demonstrated in Fig. (**1**).

Our foundational data for gut microbiota was extracted from the MiBioGen study [40, 41]. This magnum opus in metagenomic research masterfully integrated genome-wide genotyping data with 16S fecal microbiota profiles across an impressive consortium of 24 cohorts, encapsulating 18,340 individuals, predominantly of European heritage (N = 13,266). By targeting the V4, V3-V4, and V1-V2 regions of the 16S rRNA gene, the study unveiled a granular microbial landscape. Subsequent taxonomic binning revealed a diverse microbial consortium of 211 taxa [40]. In parallel, our outcome metrics were anchored to the Finngen study (https://r7.finngen.fi/) (GWAS ID: finn-b-ST19_POISO_ NARCOT_PSYCHOD_HALLUCINOG), a comprehensive dataset encompassing 270 cases and 213,568 controls, underpinned by a staggering 16,380,433 SNPs [42].

### 2.2 Genetic Instruments Extraction

The confluence of narcotics, psychodysleptics, and gut microbial communities has catalyzed fervent discussions in the Mendelian randomization arena. To underpin our analyses with precision, we embarked on a meticulous journey of genetic instrument extraction. Genetic variants were sieved through rigorous criteria: a *p*-value threshold below 1×10^-5^, ensuring robust associations with gut microbiota, and an r^2^ threshold below 0.001 to obviate linkage disequilibrium concerns [43]. These vetted variants were subsequently harmonized with SNP outcomes associated with “Poisoning by narcotics and psychodysleptics (hallucinogens)”.

### 2.3 Mendelian Randomization

Armed with our curated datasets, our focus shifted to genetic instruments intrinsically linked to gut microbiota variations. These instruments, emblematic of gut microbiota composition, were shielded from extraneous confounding variables. Our MR analytical machinery was then set in motion, with the overarching objective of discerning the causal nexus between gut microbiota and the risk trajectory of poisoning by narcotics and psychodysleptics, anchored by the inverse-variance weighted (IVW) method. This approach is particularly effective in two-sample MR analyses for uncovering causal relationships. To validate our findings, we compared the results from the IVW method with those obtained using the weighted median and MR-Egger methods. The weighted median approach is designed to minimize the influence of potentially invalid IVs, ensuring they don't impact the results by more than 50%. On the other hand, the MR-Egger method is useful in situations where all IVs might be invalid, providing an additional layer of verification.

Furthermore, to enhance the accuracy of our analysis, we incorporated the False Discovery Rate (FDR) correction. This statistical adjustment is crucial in controlling the rate of type I errors, particularly when dealing with multiple comparisons. The MR effect estimate was deemed statistically significant with a false discovery rate (FDR) of less than 10%. The Benjamini-Hochberg method was applied to adjust for the multiple taxa tested, considering multiple comparisons. By applying the FDR correction, we aimed to ensure that our findings were not only statistically significant but also reliable and less likely to be influenced by random chance. This additional step in our methodology underscores our commitment to delivering precise and trustworthy results in our exploration of the complex interactions between gut microbiota and drug poisoning risks.

### 2.4 Sensitivity Assessment

To further validate the reliability of our findings, we conducted comprehensive evaluations addressing concerns of multiplicity. This included rigorous testing for variance among the different instrumental variables and assessing the impact of multiple statistical tests. We meticulously evaluated heterogeneity within the IVW models using Cochran's Q test, where a *p*-value below 0.05 indicated significant heterogeneity. Although heterogeneity does not invalidate the IVW model, it necessitates additional analytical scrutiny. The MR-Egger method played a critical role in our analysis, particularly in detecting and adjusting for directional pleiotropy, as indicated by its non-zero intercept [44]. We also performed leave-one-out analyses to gauge the impact of individual SNPs on the overall results and used the MR-PRESSO method for robust outlier detection. Any identified outliers were promptly removed, followed by a reanalysis of the MR data to ensure the accuracy and integrity of our study.

MR evaluations were conducted using the R packages “TwoSampleMR” (version 0.5.6), “MRcML” (version 0.0.0.9), and “MendelianRandomization” (version 0.6.0) on R (version 4.1.2) [44].

## 3 RESULTS

### 3.1 Instrumental Variables (IVs) and Main MR Analyses

A comprehensive IVW methodological approach was employed to investigate the associations between genetically predicted gut microbiota and the risk of Poisoning by narcotics and psychodysleptics (hallucinogens). After matching with the SNPs in the outcome of “Poisoning by narcotics and psychodysleptics (hallucinogens)”, 200 microbial taxa remained. Using the IVW method, these 200 taxa were analyzed, including 16 at the class level, 31 at the family level, 110 at the genus level, 19 at the order level, 9 at the phylum level, and 15 unknown microbial taxa. Our analysis encompassed a diverse microbial taxonomy, including 16 classes, 31 families, 110 genera, 19 orders, 9 phyla, and 15 entities of unidentified classification.

**Fig. (2):**
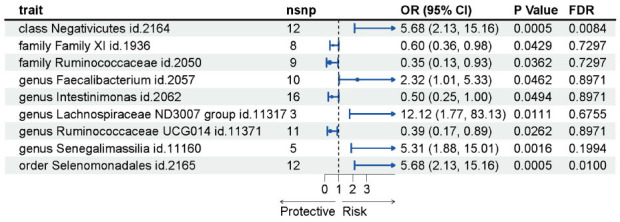
Forest plot. This plot displayed the association between various gut microbiota and outcomes. Each horizontal line represents the 95% confidence interval (CI) for the odds ratio (OR) of a specific gut microbiota on the outcome. The black dot on each line indicates the point estimate of the OR. The p values and number of SNPs used as instrumental variables for each microbiota is also provided.

From this extensive taxonomy, a subset of 9 microbial communities demonstrated statistical significance with a *p*-value less than 0.05, namely Class Negativicutes (OR 5.68, 95% CI 2.13-15.16, *p =* 0.0005), Family *XI* (OR 0.60, 95% CI 0.36-0.98, *p =* 0.0429), Family Ruminococcaceae (OR 0.35, 95% CI 0.13-0.93, *p =* 0.0362), Genus *Faecalibacterium* (OR 2.32, 95%CI 1.01-5.33, *p =* 0.0462), Genus *Intestinimonas* (OR 0.50, 95% CI 0.25-1.00, *p =* 0.0494), Genus *Lachnospiraceae* ND3007 group (OR 12.12, 95% CI 1.77-83.13, *p =* 0.0111), Genus *Ruminococcaceae UCG014* (OR 0.39, 95% CI 0.17-0.89, *p =* 0.0262), Genus *Senegalimassilia* (OR 5.31, 95% CI 1.88-15.01, *p =* 0.0016), Order *Selenomonadales* (OR 5.68, 95% CI 2.13-15.16, *p =* 0.0005) (Fig. **2**).

Notably, after applying FDR multiple corrections, a pronounced causal association was identified between the outcome and both Class Negativicutes and Order Selenomonadales. It is worth mentioning that both the Class Negativicutes and Order Selenomonadales are taxonomically synonymous, leading to congruent findings in our analysis. The detailed associations of these microbial communities with the outcome are presented in Table **1**.

### 3.2 Heterogeneity and Pleiotropy Assessment

To assess the heterogeneity among the instrumental variables, Cochran’s Q test was employed. The results indicated that among the nine microbial communities with *p*-values less than 0.05, no significant heterogeneity was observed (Figs. **S1**-**S3**).

Furthermore, to explore the potential pleiotropy of the instrumental variables, we utilized the intercept term of the MR Egger method. Our findings did not suggest any significant pleiotropic effects. The detailed results of the heterogeneity and pleiotropy assessments are provided in Table **S1**.

### 3.3 Sensitivity Analyses

To ensure the robustness of our findings, we conducted sensitivity analyses using various methods, including Weighted median, MR PRESSO, Maximum likelihood, and IVW radial. Across all these analyses, the results consistently indicated a significant association with the microbial community. This consistency underscores the robustness and reliability of our research outcomes. The detailed results of the sensitivity analyses are presented in Table **S2**.

## 4 DISCUSSION

The intricate relationship between gut microbiota and human health has increasingly come under the scientific spotlight. Through our Mendelian randomization lens, we present compelling data that draws a causal thread between specific gut microbial communities and the risk of poisoning by narcotics and psychodysleptics. Within the vast microbial mosaic, our findings emphasize key taxa: Negativicutes, Family XI, Ruminococcaceae, Faecalibacterium, Intestinimonas, Lachnospiraceae ND3007 group, Ruminococcaceae UCG014, Senegalimassilia, and Selenomonadales. Yet, the intricate mechanistic underpinnings connecting these microbial players to poisoning events associated with narcotics and psychodysleptics beckon further elucidation. Notably, to our knowledge, this is the first MR study exploring the relationship between gut microbiota and poisoning by narcotics and psychodysleptics.

Previous studies have extensively examined the impact of narcotics, particularly opioids, on the gut microbiota. Chronic morphine use has been shown to significantly alter the gut microbiota, increasing Gram-positive pathogens and affecting bile acid levels. Restoring the microbiome can counteract these imbalances, suggesting new treatment approaches for morphine users [45]. Morphine use leads to shifts in the gut microbiome and metabolome, contributing to opioid-related adverse effects. In germ-free and antibiotic-treated mice, morphine tolerance is reduced, but reintroducing specific bacteria restores it, indicating that morphine disrupts the gut balance and triggers inflammation and tolerance [46]. Probiotics might help in balancing these effects [47].

Morphine doesn't directly change the intestinal microbiota, but its effects might be mediated through specific receptors, affecting gastrointestinal transit, gut barrier integrity, and bile circulation [48, 49]. MR analysis, using genetic variations, helps in understanding these causal relationships, offering a more detailed view of the complex interactions between drugs and the gut microbiome.

### 4.1 Drug Metabolism

The gut microbiota plays a pivotal role in drug metabolism, with variations in its composition influencing both drug efficacy and toxicity [27]. Recent research suggests that the gut microbiota can influence drug metabolism, potentially altering the pharmacokinetics and pharmacodynamics of certain medications. This microbial-mediated drug metabolism can lead to variations in drug efficacy and safety profiles. For instance, specific bacteria in the gut can activate, inactivate, or even toxify certain drugs, leading to therapeutic outcomes that differ from what is anticipated. In some cases, this can result in what would be a normally functioning drug producing heightened adverse reactions, effectively leading to poisoning. This highlights the importance of considering individual gut microbiota compositions when prescribing medications. As personalized medicine continues to evolve, understanding the interplay between gut microbiota and drug metabolism will be crucial in optimizing therapeutic strategies and minimizing adverse drug reactions.

### 4.2 Gut-brain Axis

The bidirectional communication between the gastrointestinal tract and the central nervous system, known as the gut-brain axis, has attracted significant attention in recent years. This communication, facilitated by neural, endocrine, immune, and metabolic pathways, not only plays a pivotal role in maintaining physiological homeostasis [50]. Xie *et al*.'s findings underscore the vital role of the gut-brain axis in defending against toxins and highlight its shared neural response with drugs [51]. The study serves as a foundational step in this direction, emphasizing the need for a holistic understanding of the gut-brain axis in the context of toxin exposure and drug-induced adverse reactions.

The influence of opioid on the gut-brain axis has been elucidated, revealing potential dysbiosis and subsequent alterations [52]. This disruption to the gut microbiota's equilibrium can have cascading effects on the central nervous system. A tripartite relationship emerges when considering gut microbiota, immunity, and pain [53]. The gut microbiota emerges as a critical modulator of immune responses, which subsequently can shape pain perception. Interactions between NSAIDs, opioids, and the gut microbiota present novel avenues for therapeutic strategies in managing inflammation and pain [54].

### 4.3 Addiction

As for narcotics and psychodysleptics, addiction poses a significant concern. The realm of addiction research has also been enriched with insights into the potential role of the gut microbiota-brain axis in drug addiction [55]. The gut microbiota's profound influence on neurobiological pathways associated with addiction becomes evident. Mapping gut microbiota changes reveals associations with morphine abstinence-induced depressive behaviors [56]. A nexus between morphine withdrawal, gut microbiota alterations, and depressive symptoms becomes discernible. The therapeutic synergy of cannabinoids and opioids, especially in the context of inflammatory bowel diseases, has been underscored [57]. This synergy might be mediated through interactions with the gut microbiota.

*Negativicoccus* and Selenomonadales are part of the Firmicutes phylum, commonly found in the human gut. They play roles in fermenting organic acids, contributing to gut homeostasis, and potentially influencing various physiological processes. Novel techniques have identified specific conserved signature indels (CSIs) for all species within the Selenomonadales [58]. Recent investigations have reported a conspicuous reduction of Selenomonadales in the gut microbiome of patients diagnosed with acute myocardial infarction (AMI) [59]. While the mechanistic underpinnings remain to be fully elucidated, it is hypothesized that perturbations in the gut microbiota, including the diminution of Selenomonadales, may influence systemic inflammatory cascades and immune responses, the key determinants of cardiovascular integrity [60, 61]. Concurrently, certain narcotics and psychodysleptics, such as cocaine, are known to amplify the risk of cardiovascular incidents [56, 57]. This posits a complex interplay wherein narcotics might exert dual effects: a direct impact on cardiovascular health and an indirect influence mediated by gut microbiota alterations, including shifts in *Selenomonadales* prevalence. As we navigate this intricate landscape, it becomes paramount to disentangle the overt cardiovascular ramifications of narcotics from the nuanced effects mediated by the gut microbiota.

*Negativicoccus*, a genus under the Order Selenomonadales, represents a previously identified species found in the fecal sample of an obese individual post-bariatric surgery [62, 63]. *Negativicutes* emerge as a pivotal player, notably augmenting propionate production [64]. Propionate, a short-chain fatty acid, has been implicated in modulating hepatic drug metabolism processes, particularly in opioids like Fentanyl [65]. Key hepatic enzymes, such as those from the cytochrome P450 family, instrumental in drug metabolism, may be regulated by propionate levels [66]. Such metabolic alterations could influence the bioavailability, efficacy, and toxicity of drugs. Specifically, for commonly administered substances like narcotics and psychodysleptics, their *in vivo* concentrations and activities might be perturbed. This could potentially escalate the risks associated with narcotic poisoning and psychodysleptic effects, especially in individuals where the gut microbiota deviates from the norm or is influenced by external factors like diet, medications, or underlying conditions.

The inverse association of *Intestinimonas*, *Ruminococcaceae*, and Family XI with the outcome of poisoning by narcotics and psychodysleptics suggests a potential protective role of these bacterial taxa. *Intestinimonas* is known for its butyrate-producing capabilities [67]. Butyrate is a short-chain fatty acid that has been linked to anti-inflammatory effects and improved gut barrier function. Enhancing the gut barrier, it may limit the absorption of harmful substances, including narcotics and psychodysleptics. Ruminococcaceae, a diverse family within the Firmicutes phylum, has been associated with the production of anti-inflammatory compounds and the modulation of T-cell responses [68, 69]. A balanced immune response can mitigate the systemic effects of toxins and drugs.

## 5 LIMITATIONS

In our MR study, we explore the relationship between gut microbiota and the effects of narcotics and psychodysleptics, offering potential therapeutic and prescribing insights. However, the study's validity relies heavily on the instrumental variables used, and any deviations from MR assumptions could lead to biases, especially due to horizontal pleiotropy risks. Our findings, primarily from European populations, may have limited global applicability. While MR sheds light on causality, it doesn't replace the comprehensive insights provided by randomized controlled trials (RCTs), which are essential for further validation and exploration. Additionally, the gut microbiota is a complex ecosystem, and there are likely many intricate mechanisms of interaction that remain to be explored. Our study is an initial step, and more foundational experiments are needed to validate and expand upon these findings. The gut microbiota's role in drug effects is a vast area of study, and our research serves as a starting point for more extensive, diverse investigations into this complex relationship.

## CONCLUSION

The interplay between gut microbiota and the human response to narcotics and psychodysleptics is a burgeoning field. Our study, using robust MR analyses, reveals specific microbial taxa with potential roles in poisoning by narcotics and psychodysleptics. These findings highlight the gut microbiota's potential as both a therapeutic target and predictive tool in this context. While our research is a stepping stone, it paves the way for future investigations that may transform our understanding of drug responses and open the door to personalized medicine guided by an individual's unique microbial signature.

## Figures and Tables

**Fig. (1):**
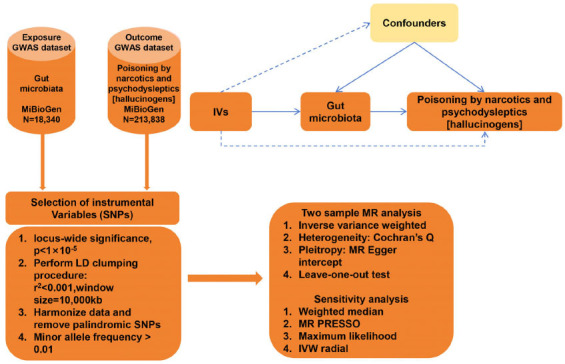
Flowchart of the present MR study and major assumptions.

**Table 1:** Causal effect of gut microbiota on poisoning by narcotics and psychodysleptics (GWAS ID: finn-b-ST19_POISO_ NARCOT_PSYCHOD_HALLUCINOG).

**Trait**	**nsnp**	**OR (95%CI)**	***P* value**	**fdr**
Class Negativicutes id.2164	12	5.68(2.13,15.16)	0.0005	0.0084
Family Family XI id.1936	8	0.60(0.36,0.98)	0.0429	0.7297
Family Ruminococcaceae id.2050	9	0.35(0.13,0.93)	0.0362	0.7297
Genus *Faecalibacterium* id.2057	10	2.32(1.01,5.33)	0.0462	0.8971
Genus *Intestinimonas* id.2062	16	0.50(0.25,1.00)	0.0494	0.8971
Genus *Lachnospiraceae* ND3007 group id.11317	3	12.12(1.77,83.13)	0.0111	0.6755
Genus *Ruminococcaceae* UCG014 id.11371	11	0.39(0.17,0.89)	0.0262	0.8971
Genus *Senegalimassilia* id.11160	5	5.31(1.88,15.01)	0.0016	0.1994
Order Selenomonadales id.2165	12	5.68(2.13,15.16)	0.0005	0.0100

## Data Availability

The data that supports the findings of this study are available in the main text and materials of this article.

## LIST OF ABBREVIATIONS

Csis: Conserved Signature Indels
FDR: False Discovery Rate
IVW: Inverse-variance Weighted
LSD: Lysergic Acid Diethylamide
MR: Mendelian Randomization
PTSD: Post-traumatic Stress Disorder
Rcts: Randomized Controlled Trials

## STANDARDS OF REPORTING

STROBE guidelines were followed.

## References

[1] Foley K.M. (1993). Opioids.. Neurol. Clin..

[2] Johannes C.B., Le T.K., Zhou X., Johnston J.A., Dworkin R.H. (2010). The prevalence of chronic pain in United States adults: Results of an Internet-based survey.. J. Pain.

[3] Stein C. (2018). New concepts in opioid analgesia.. Expert Opin. Investig. Drugs.

[4] Nafziger A.N., Barkin R.L. (2018). Opioid therapy in acute and chronic pain.. J. Clin. Pharmacol..

[5] Vollenweider F.X. (2001). Brain mechanisms of hallucinogens and entactogens.. Dialogues Clin. Neurosci..

[6] Volgin A.D., Yakovlev O.A., Demin K.A., Alekseeva P.A., Kyzar E.J., Collins C., Nichols D.E., Kalueff A.V. (2019). Understanding central nervous system effects of deliriant hallucinogenic drugs through experimental animal models.. ACS Chem. Neurosci..

[7] Liechti M.E. (2017). Modern clinical research on LSD.. Neuropsychopharmacology.

[8] Ling S., Ceban F., Lui L.M.W., Lee Y., Teopiz K.M., Rodrigues N.B., Lipsitz O., Gill H., Subramaniapillai M., Mansur R.B., Lin K., Ho R., Rosenblat J.D., Castle D., McIntyre R.S. (2022). Molecular mechanisms of psilocybin and implications for the treatment of depression.. CNS Drugs.

[9] Kaye A.D., Jones M.R., Kaye A.M., Ripoll J.G., Galan V., Beakley B.D., Calixto F., Bolden J.L., Urman R.D., Manchikanti L. (2017). Prescription opioid abuse in chronic pain: An updated review of opioid abuse predictors and strategies to curb opioid abuse: Part 1.. Pain Physician.

[10] Brady K.T., McCauley J.L., Back S.E. (2016). Prescription opioid misuse, abuse, and treatment in the United States: An update.. Am. J. Psychiatry.

[11] Vearrier D., Grundmann O. (2021). Clinical pharmacology, toxicity, and abuse potential of opioids.. J. Clin. Pharmacol..

[12] Nichols D.E., Grob C.S. (2018). Is LSD toxic?. Forensic Sci. Int..

[13] Hardaway R., Schweitzer J., Suzuki J. (2016). Hallucinogen use disorders.. Child Adolesc. Psychiatr. Clin. N. Am..

[14] Dart R.C., Surratt H.L., Cicero T.J., Parrino M.W., Severtson S.G., Bucher-Bartelson B., Green J.L. (2015). Trends in opioid analgesic abuse and mortality in the United States.. N. Engl. J. Med..

[15] Paulozzi L.J., Budnitz D.S., Xi Y. (2006). Increasing deaths from opioid analgesics in the United States.. Pharmacoepidemiol. Drug Saf..

[16] Boyer E.W. (2012). Management of opioid analgesic overdose.. N. Engl. J. Med..

[17] Wang L., Wu Y., Yin P., Cheng P., Liu Y., Schwebel D.C., Qi J., Ning P., Liu J., Cheng X., Zhou M., Hu G. (2018). Poisoning deaths in China, 2006–2016.. Bull. World Health Organ..

[18] Inocencio T.J., Carroll N.V., Read E.J., Holdford D.A. (2013). The economic burden of opioid-related poisoning in the United States.. Pain Med..

[19] Adak A., Khan M.R. (2019). An insight into gut microbiota and its functionalities.. Cell. Mol. Life Sci..

[20] Gill S.R., Pop M., DeBoy R.T., Eckburg P.B., Turnbaugh P.J., Samuel B.S., Gordon J.I., Relman D.A., Fraser-Liggett C.M., Nelson K.E. (2006). Metagenomic analysis of the human distal gut microbiome.. Science.

[21] Fan Y., Pedersen O. (2021). Gut microbiota in human metabolic health and disease.. Nat. Rev. Microbiol..

[22] Wang Z., Wang Y., Xiong J., Gan X., Bao Y., Jiang A., Zhou Y., Huangfu Z., Yang Y., Liu Z., Xia D., Wang L. (2023). Causal effects of hypertension on risk of erectile dysfunction: A two-sample Mendelian randomization study.. Front. Cardiovasc. Med..

[23] Michaudel C., Sokol H. (2020). The Gut Microbiota at the Service of Immunometabolism.. Cell Metab..

[24] Honda K., Littman D.R. (2016). The microbiota in adaptive immune homeostasis and disease.. Nature.

[25] Järbrink-Sehgal E., Andreasson A. (2020). The gut microbiota and mental health in adults.. Curr. Opin. Neurobiol..

[26] Li H., He J., Jia W. (2016). The influence of gut microbiota on drug metabolism and toxicity.. Expert Opin. Drug Metab. Toxicol..

[27] Kang M.J., Kim H.G., Kim J.S., Oh D.G., Um Y.J., Seo C.S., Han J.W., Cho H.J., Kim G.H., Jeong T.C., Jeong H.G. (2013). The effect of gut microbiota on drug metabolism.. Expert Opin. Drug Metab. Toxicol..

[28] Thanassoulis G., O’Donnell C.J. (2009). Mendelian Randomization.. JAMA.

[29] Davies N.M., Holmes M.V., Davey Smith G. (2018). Reading Mendelian randomisation studies: a guide, glossary, and checklist for clinicians.. BMJ.

[30] Davey Smith G., Paternoster L., Relton C. (2017). When will mendelian randomization become relevant for clinical practice and public health?. JAMA.

[31] Li N., Wang Y., Wei P., Min Y., Yu M., Zhou G., Yuan G., Sun J., Dai H., Zhou E., He W., Sheng M., Gao K., Zheng M., Sun W., Zhou D., Zhang L. (2023). Causal effects of specific gut microbiota on chronic kidney diseases and renal function—a two-sample mendelian randomization study.. Nutrients.

[32] Jin Q., Ren F., Dai D., Sun N., Qian Y., Song P. (2023). The causality between intestinal flora and allergic diseases: Insights from a bi-directional two-sample Mendelian randomization analysis.. Front. Immunol..

[33] Yang M., Luo P., Zhang F., Xu K., Feng R., Xu P. (2022). Large-scale correlation analysis of deep venous thrombosis and gut microbiota.. Front. Cardiovasc. Med..

[34] Luo S., Li W., Li Q., Zhang M., Wang X., Wu S., Li Y. (2023). Causal effects of gut microbiota on the risk of periodontitis: a two-sample Mendelian randomization study.. Front. Cell. Infect. Microbiol..

[35] Liu D., Bu D., Li H., Wang Q., Ding X., Fang X. (2023). Intestinal metabolites and the risk of autistic spectrum disorder: A two-sample Mendelian randomization study.. Front. Psychiatry.

[36] Wang F., Li N., Ni S., Min Y., Wei K., Sun H., Fu Y., Liu Y., Lv D. (2023). The effects of specific gut microbiota and metabolites on IgA nephropathy-based on mendelian randomization and clinical validation.. Nutrients.

[37] Li Y., Fu R., Li R., Zeng J., Liu T., Li X., Jiang W. (2023). Causality of gut microbiome and hypertension: A bidirectional mendelian randomization study.. Front. Cardiovasc. Med..

[38] Zeng Y., Cao S., Yang H. (2023). Roles of gut microbiome in epilepsy risk: A Mendelian randomization study.. Front. Microbiol..

[39] Skrivankova V.W., Richmond R.C., Woolf B.A.R., Yarmolinsky J., Davies N.M., Swanson S.A., VanderWeele T.J., Higgins J.P.T., Timpson N.J., Dimou N., Langenberg C., Golub R.M., Loder E.W., Gallo V., Tybjaerg-Hansen A., Davey Smith G., Egger M., Richards J.B. (2021). Strengthening the reporting of observational studies in epidemiology using mendelian randomization.. JAMA.

[40] Kurilshikov A., Medina-Gomez C., Bacigalupe R., Radjabzadeh D., Wang J., Demirkan A., Le Roy C.I., Raygoza Garay J.A., Finnicum C.T., Liu X., Zhernakova D.V., Bonder M.J., Hansen T.H., Frost F., Rühlemann M.C., Turpin W., Moon J.Y., Kim H.N., Lüll K., Barkan E., Shah S.A., Fornage M., Szopinska-Tokov J., Wallen Z.D., Borisevich D., Agreus L., Andreasson A., Bang C., Bedrani L., Bell J.T., Bisgaard H., Boehnke M., Boomsma D.I., Burk R.D., Claringbould A., Croitoru K., Davies G.E., van Duijn C.M., Duijts L., Falony G., Fu J., van der Graaf A., Hansen T., Homuth G., Hughes D.A., Ijzerman R.G., Jackson M.A., Jaddoe V.W.V., Joossens M., Jørgensen T., Keszthelyi D., Knight R., Laakso M., Laudes M., Launer L.J., Lieb W., Lusis A.J., Masclee A.A.M., Moll H.A., Mujagic Z., Qibin Q., Rothschild D., Shin H., Sørensen S.J., Steves C.J., Thorsen J., Timpson N.J., Tito R.Y., Vieira-Silva S., Völker U., Völzke H., Võsa U., Wade K.H., Walter S., Watanabe K., Weiss S., Weiss F.U., Weissbrod O., Westra H.J., Willemsen G., Payami H., Jonkers D.M.A.E., Arias Vasquez A., de Geus E.J.C., Meyer K.A., Stokholm J., Segal E., Org E., Wijmenga C., Kim H.L., Kaplan R.C., Spector T.D., Uitterlinden A.G., Rivadeneira F., Franke A., Lerch M.M., Franke L., Sanna S., D’Amato M., Pedersen O., Paterson A.D., Kraaij R., Raes J., Zhernakova A. (2021). Large-scale association analyses identify host factors influencing human gut microbiome composition.. Nat. Genet..

[41] Consortium M. (2022). MiBioGen. https://mibiogen.gcc.rug.nl/.

[42] FinnGen (2022). ST19_POISO_NARCOT_PSYCHOD_HALLUCINOG – Poisoning by narcotics and psychodysleptics (hallucinogens). Risteys v2.1.0.. https://risteys.finregistry.fi/endpoints/ST19_POISO_NARCOT_PSYCHOD_HALLUCINOG.

[43] Pierce B.L., Ahsan H., VanderWeele T.J. (2011). Power and instrument strength requirements for Mendelian randomization studies using multiple genetic variants.. Int. J. Epidemiol..

[44] Verbanck M., Chen C.Y., Neale B., Do R. (2018). Detection of widespread horizontal pleiotropy in causal relationships inferred from Mendelian randomization between complex traits and diseases.. Nat. Genet..

[45] Wang F., Meng J., Zhang L., Johnson T., Chen C., Roy S. (2018). Morphine induces changes in the gut microbiome and metabolome in a morphine dependence model.. Sci. Rep..

[46] Banerjee S., Sindberg G., Wang F., Meng J., Sharma U., Zhang L., Dauer P., Chen C., Dalluge J., Johnson T., Roy S. (2016). Opioid-induced gut microbial disruption and bile dysregulation leads to gut barrier compromise and sustained systemic inflammation.. Mucosal Immunol..

[47] Zhang L., Meng J., Ban Y., Jalodia R., Chupikova I., Fernandez I., Brito N., Sharma U., Abreu M.T., Ramakrishnan S., Roy S. (2019). Morphine tolerance is attenuated in germfree mice and reversed by probiotics, implicating the role of gut microbiome.. Proc. Natl. Acad. Sci. USA.

[48] Wang F., Roy S. (2017). Gut Homeostasis, Microbial Dysbiosis, and Opioids.. Toxicol. Pathol..

[49] Akbarali H.I., Dewey W.L. (2019). Gastrointestinal motility, dysbiosis and opioid-induced tolerance: is there a link?. Nat. Rev. Gastroenterol. Hepatol..

[50] Mayer E.A., Nance K., Chen S. (2022). The Gut–Brain Axis.. Annu. Rev. Med..

[51] Xie Z., Zhang X., Zhao M., Huo L., Huang M., Li D., Zhang S., Cheng X., Gu H., Zhang C., Zhan C., Wang F., Shang C., Cao P. (2022). The gut-to-brain axis for toxin-induced defensive responses.. Cell.

[52] Rueda-Ruzafa L., Cruz F., Cardona D., Hone A.J., Molina-Torres G., Sánchez-Labraca N., Roman P. (2020). Opioid system influences gut-brain axis: Dysbiosis and related alterations.. Pharmacol. Res..

[53] Santoni M., Miccini F., Battelli N. (2021). Gut microbiota, immunity and pain.. Immunol. Lett..

[54] Zádori Z.S., Király K., Al-Khrasani M., Gyires K. (2023). Interactions between NSAIDs, opioids and the gut microbiota - Future perspectives in the management of inflammation and pain.. Pharmacol. Ther..

[55] Luo X., Li H., Fan X., Wu X., Zhou R., Lei Y., Xue D., Yang F., Xu Y., Wang K. (2023). The gut microbiota-brain Axis: Potential mechanism of drug addiction.. Curr. Top. Med. Chem..

[56] Ji J., Yan N., Zhang Z., Li B., Xue R., Dang Y. (2022). Characterized profiles of gut microbiota in morphine abstinence-induced depressive-like behavior.. Neurosci. Lett..

[57] Kienzl M., Storr M., Schicho R. (2020). Cannabinoids and opioids in the treatment of inflammatory bowel diseases.. Clin. Transl. Gastroenterol..

[58] Campbell C., Adeolu M., Gupta R.S. (2015). Genome-based taxonomic framework for the class Negativicutes: division of the class Negativicutes into the orders Selenomonadales emend., Acidaminococcales ord. nov. and Veillonellales ord. nov.. Int. J. Syst. Evol. Microbiol..

[59] Chiu F.C., Tsai C.F., Huang P.S., Shih C.Y., Tsai M.H., Hwang J.J., Wang Y.C., Chuang E.Y., Tsai C.T., Chang S.N. (2022). The gut microbiome, seleno-compounds, and acute myocardial infarction.. J. Clin. Med..

[60] Chen H., Jia Z., He M., Chen A., Zhang X., Xu J., Wang C. (2023). Arula-7 powder improves diarrhea and intestinal epithelial tight junction function associated with its regulation of intestinal flora in calves infected with pathogenic Escherichia coli O1.. Microbiome.

[61] Song P., Yang D., Wang H., Cui X., Si X., Zhang X., Zhang L. (2020). Relationship between intestinal flora structure and metabolite analysis and immunotherapy efficacy in Chinese NSCLC patients.. Thorac. Cancer.

[62] Togo A.H., Khelaifia S., Valero R., Cadoret F., Raoult D., Million M. (2016). ‘Negativicoccus massiliensis’, a new species identified from human stool from an obese patient after bariatric surgery.. New Microbes New Infect..

[63] Marchandin H., Teyssier C., Campos J., Jean-Pierre H., Roger F., Gay B., Carlier J.P., Jumas-Bilak E. (2010). Negativicoccus succinicivorans gen. nov., sp. nov., isolated from human clinical samples, emended description of the family Veillonellaceae and description of Negativicutes classis nov., Selenomonadales ord. nov. and Acidaminococcaceae fam. nov. in the bacterial phylum Firmicutes.. Int. J. Syst. Evol. Microbiol..

[64] Reichardt N., Duncan S.H., Young P., Belenguer A., McWilliam Leitch C., Scott K.P., Flint H.J., Louis P. (2014). Phylogenetic distribution of three pathways for propionate production within the human gut microbiota.. ISME J..

[65] Ziesenitz V.C., Vaughns J.D., Koch G., Mikus G., van den Anker J.N. (2018). Pharmacokinetics of fentanyl and its derivatives in children: A comprehensive review.. Clin. Pharmacokinet..

[66] Perry R.J., Borders C.B., Cline G.W., Zhang X.M., Alves T.C., Petersen K.F., Rothman D.L., Kibbey R.G., Shulman G.I. (2016). Propionate increases hepatic pyruvate cycling and anaplerosis and alters mitochondrial metabolism.. J. Biol. Chem..

[67] Louis P., Flint H.J. (2009). Diversity, metabolism and microbial ecology of butyrate-producing bacteria from the human large intestine.. FEMS Microbiol. Lett..

[68] Sinha S.R., Haileselassie Y., Nguyen L.P., Tropini C., Wang M., Becker L.S., Sim D., Jarr K., Spear E.T., Singh G., Namkoong H., Bittinger K., Fischbach M.A., Sonnenburg J.L., Habtezion A. (2020). Dysbiosis-induced secondary bile acid deficiency promotes intestinal inflammation.. Cell Host Microbe.

[69] Keshteli A., Valcheva R., Nickurak C., Park H., Mandal R., van Diepen K., Kroeker K., van Zanten S., Halloran B., Wishart D., Madsen K., Dieleman L. (2022). Anti-inflammatory diet prevents subclinical colonic inflammation and alters metabolomic profile of ulcerative colitis patients in clinical remission.. Nutrients.

