# Supplementary material for: Deciphering the Causality between Gut Microbiota Dysbiosis and Poisoning by Narcotics and Psychodysleptics: A Mendelian Randomization Analysis
Source: Curr Neuropharmacol. 2024 Jul 30;23(2):187–95. doi: 10.2174/1570159X22999240729092453 (PMC11793043; doi:10.2174/1570159X22999240729092453)
Supplement: Supplementary file 1 [file CN-23-2-187_SD1.pdf]

Supplementary Material

Deciphering the Causality between Gut Microbiota Dysbiosis and Poisoning by Narcotics and Psychodysleptics: A Mendelian Randomization Analysis

Ning Wang[1,2] and Zhenbo Su[1,*]

[1]Department of Anesthesiology, China-Japan Union Hospital of Jilin University, Changchun, China; [2]Department of Anesthesiology, Shanghai Ruijin Hospital, Shanghai, China

1875-6190/25 $65.00+.00 © 2025 Bentham Science Publishers

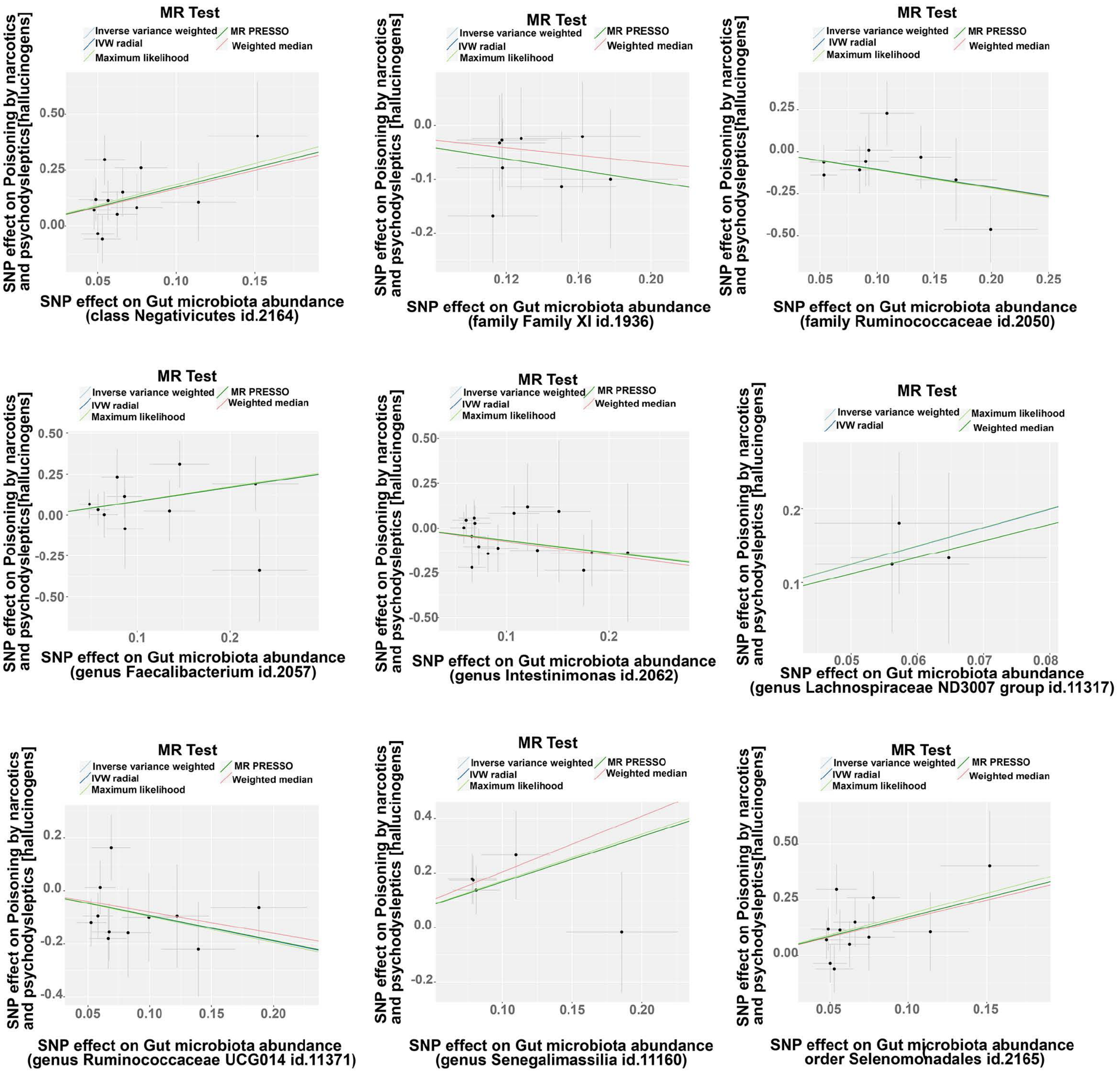

Fig. (S1). Scatter Plot of Sensitivity Analysis. The horizontal axis represents the effect strength between the SNP and the exposure, while the vertical axis represents the effect strength between the SNP and the outcome. The black dots indicate the effect values of the SNP with exposure and outcome, and the solid line represents the causal association between the microbiota and outcome. Different colors represent different MR methods.

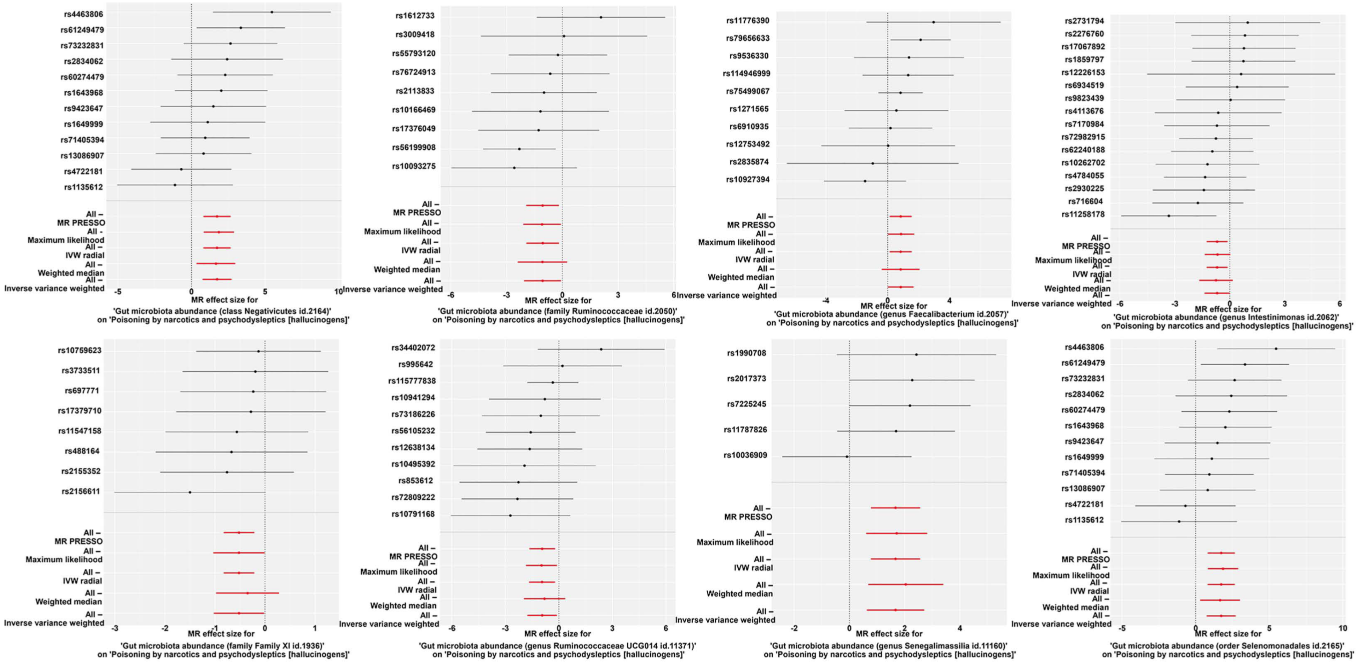

Fig. (S2). Forest Plot. A leave-one-out sensitivity test was further conducted to determine whether the causal estimate was driven by any single SNP, revealing a consistent positive correlation between the genetically predicted microbiota and the outcome. The genus Lachnospiraceae ND3007 group id.11317 was not included in the leave-one-out sensitivity test due to the limited number of instrumental variables (IVs).

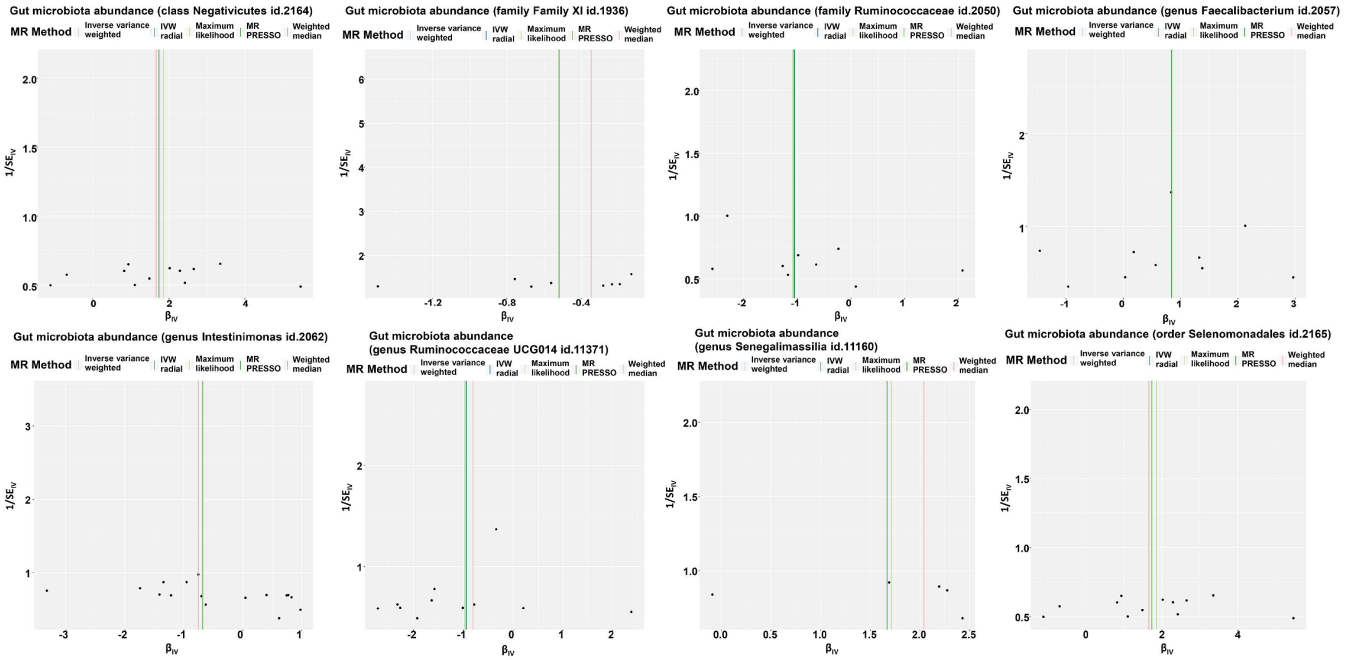

Fig. (S3). Heterogeneity Analysis. Instrumental variables from different analysis platforms, experiments, and populations may exhibit heterogeneity, which can affect the results of Mendelian Randomization analysis. Heterogeneity is assessed using IVW and MR-Egger tests, with a p-value <0.05 indicating the presence of heterogeneity in the study. The genus Lachnospiraceae ND3007 group id.11317, due to a limited number of IVs, is not included in the heterogeneity analysis.

Table S1. Evaluation of heterogeneity and directional pleiotropy using different methods.

Trait	nsnp	Q	Q_pval	egger_intercept	se	P value
Class Negativicutes id.2164	12	9.7073	0.5569	-0.0632	0.1104	0.5798
Family Family XI id.1936	8	2.5864	0.9205	-0.0972	0.2161	0.6686
Family Ruminococcaceae id.2050	9	6.2340	0.6210	-0.0059	0.1085	0.9584
Genus Faecalibacterium id.2057	10	6.4925	0.6898	0.0396	0.0858	0.6565
Genus Intestinimonas id.2062	16	10.2300	0.8050	0.0172	0.0835	0.8393
Genus Lachnospiraceae ND3007 group id.11317	3	0.2430	0.8856	0.2281	0.9703	0.8530
Genus Ruminococcaceae UCG014 id.11371	11	7.6215	0.6658	-0.0704	0.0853	0.4307
Genus Senegalimassilia id.11160	5	2.9329	0.5691	0.2654	0.1846	0.2461
Order Selenomonadales id.2165	12	9.7073	0.5569	-0.0632	0.1104	0.5798

Table S2. Sensitivity Analysis Results for Mendelian Randomization Associations between Gut Microbiota and Poisoning by Narcotics and Psychodysleptics

Trait	Method	nsnp	b	se	OR(95%CI)	P value
class Negativicutes id.2164	Weighted median	12	1.6592	0.6592	5.26(1.44,19.13)	0.0118
class Negativicutes id.2164	Inverse variance weighted	12	1.7367	0.5009	5.68(2.13,15.16)	0.0005
class Negativicutes id.2164	MR PRESSO	12	1.7367	0.4706	5.68(2.26,14.28)	0.0036
class Negativicutes id.2164	Maximum likelihood	12	1.8605	0.5244	6.43(2.3,17.96)	0.0004
class Negativicutes id.2164	IVW radial	12	1.7344	0.4712	5.67(2.25,14.27)	0.0002
family Family XI id.1936	Weighted median	8	-0.3468	0.3144	0.71(0.38,1.31)	0.2701
family Family XI id.1936	Inverse variance weighted	8	-0.5190	0.2564	0.6(0.36,0.98)	0.0429
family Family XI id.1936	MR PRESSO	8	-0.5190	0.1558	0.6(0.44,0.81)	0.0126
family Family XI id.1936	Maximum likelihood	8	-0.5201	0.2610	0.59(0.36,0.99)	0.0463
family Family XI id.1936	IVW radial	8	-0.5192	0.1558	0.59(0.44,0.81)	0.0009
family Ruminococcaceae id.2050	Weighted median	9	-1.0704	0.7033	0.34(0.09,1.36)	0.1280
family Ruminococcaceae id.2050	Inverse variance weighted	9	-1.0610	0.5065	0.35(0.13,0.93)	0.0362
family Ruminococcaceae id.2050	MR PRESSO	9	-1.0610	0.4472	0.35(0.14,0.83)	0.0450
family Ruminococcaceae id.2050	Maximum likelihood	9	-1.0922	0.5138	0.34(0.12,0.92)	0.0335
family Ruminococcaceae id.2050	IVW radial	9	-1.0520	0.4475	0.35(0.15,0.84)	0.0187
genus Faecalibacterium id.2057	Weighted median	10	0.8388	0.6156	2.31(0.69,7.73)	0.1730
genus Faecalibacterium id.2057	Inverse variance weighted	10	0.8435	0.4231	2.32(1.01,5.33)	0.0462
genus Faecalibacterium id.2057	MR PRESSO	10	0.8435	0.3594	2.32(1.15,4.70)	0.0435
genus Faecalibacterium id.2057	Maximum likelihood	10	0.8657	0.4334	2.38(1.02,5.56)	0.0458
genus Faecalibacterium id.2057	IVW radial	10	0.8398	0.3613	2.32(1.14,4.7)	0.0201
genus Intestinimonas id.2062	Weighted median	16	-0.7524	0.4880	0.47(0.18,1.23)	0.1231
genus Intestinimonas id.2062	Inverse variance weighted	16	-0.6884	0.3502	0.5(0.25,1.00)	0.0494
genus Intestinimonas id.2062	MR PRESSO	16	-0.6884	0.2892	0.50(0.28,0.89)	0.0310
genus Intestinimonas id.2062	Maximum likelihood	16	-0.6675	0.3556	0.51(0.26,1.03)	0.0605

Trait	Method	nsnp	b	se	OR(95%CI)	P value
genus Intestinimonas id.2062	IVW radial	16	-0.6873	0.2895	0.5(0.29,0.89)	0.0176
genus Lachnospiraceae ND3007 group id.11317	Weighted median	3	2.2359	1.2024	9.35(0.89,98.75)	0.0630
genus Lachnospiraceae ND3007 group id.11317	Inverse variance weighted	3	2.4952	0.9822	12.12(1.77,83.13)	0.0111
genus Lachnospiraceae ND3007 group id.11317	Maximum likelihood	3	2.5022	1.0321	12.21(1.61,92.31)	0.0153
genus Ruminococcaceae UCG014 id.11371	Weighted median	11	-0.8030	0.5740	0.45(0.15,1.38)	0.1618
genus Ruminococcaceae UCG014 id.11371	Inverse variance weighted	11	-0.9360	0.4209	0.39(0.17,0.89)	0.0262
genus Ruminococcaceae UCG014 id.11371	MR PRESSO	11	-0.9360	0.3674	0.39(0.19,0.81)	0.0290
genus Ruminococcaceae UCG014 id.11371	Maximum likelihood	11	-0.9748	0.4439	0.38(0.16,0.90)	0.0281
genus Ruminococcaceae UCG014 id.11371	IVW radial	11	-0.9453	0.3700	0.39(0.19,0.8)	0.0106
genus Senegalimassilia id.11160	Weighted median	5	2.0405	0.6917	7.69(1.98,29.85)	0.0032
genus Senegalimassilia id.11160	Inverse variance weighted	5	1.6699	0.5302	5.31(1.88,15.01)	0.0016
genus Senegalimassilia id.11160	MR PRESSO	5	1.6699	0.4540	5.31(2.18,12.93)	0.0212
genus Senegalimassilia id.11160	Maximum likelihood	5	1.7116	0.5633	5.54(1.84,16.70)	0.0024
order Selenomonadales id.2165	Weighted median	12	1.6592	0.6613	5.26(1.44,19.21)	0.0121
order Selenomonadales id.2165	Inverse variance weighted	12	1.7367	0.5009	5.68(2.13,15.16)	0.0005
order Selenomonadales id.2165	MR PRESSO	12	1.7367	0.4706	5.68(2.26,14.28)	0.0036
order Selenomonadales id.2165	Maximum likelihood	12	1.8605	0.5244	6.43(2.30,17.96)	0.0004
order Selenomonadales id.2165	IVW radial	12	1.7344	0.4712	5.67(2.25,14.27)	0.0002
